# MutaPT: A Multi-Task Pre-Trained Transformer for Classifying State of Disorders of Consciousness Using EEG Signal

**DOI:** 10.3390/brainsci14070688

**Published:** 2024-07-10

**Authors:** Zihan Wang, Junqi Yu, Jiahui Gao, Yang Bai, Zhijiang Wan

**Affiliations:** 1 School of Information Engineering, Nanchang University, Nanchang 330031, China; 2 School of Public Policy and Administration, Nanchang University, Nanchang 330031, China; 3 Affiliated Rehabilitation Hospital, Jiangxi Medical College, Nanchang University, Nanchang 330031, China; 4 Industrial Institute of Artificial Intelligence, Nanchang University, Nanchang 330031, China

**Keywords:** disorders of consciousness, deep learning, self-supervised pre-training, transformer, DOC state classification

## Abstract

Deep learning (DL) has been demonstrated to be a valuable tool for classifying state of disorders of consciousness (DOC) using EEG signals. However, the performance of the DL-based DOC state classification is often challenged by the limited size of EEG datasets. To overcome this issue, we introduce multiple open-source EEG datasets to increase data volume and train a novel multi-task pre-training Transformer model named MutaPT. Furthermore, we propose a cross-distribution self-supervised (CDS) pre-training strategy to enhance the model’s generalization ability, addressing data distribution shifts across multiple datasets. An EEG dataset of DOC patients is used to validate the effectiveness of our methods for the task of classifying DOC states. Experimental results show the superiority of our MutaPT over several DL models for EEG classification.

## 1. Introduction 

Electroencephalography (EEG) is a non-invasive method that captures electrical activity in the brain, offering real-time data that can be crucial for understanding and enhancing the rehabilitation process [1]. In neurorehabilitation, EEG can help track the progress of recovery in patients who have suffered from strokes, traumatic brain injuries, or other neurological impairments [2]. By monitoring brain activity, clinicians can tailor rehabilitation protocols to individual needs, enhancing the effectiveness of therapeutic interventions. For patients with disorders of consciousness (DOC), such as those in a coma, vegetative state, or minimally conscious state, EEG offers critical information about their brain function [3]. It can help distinguish between different levels of consciousness, guide decisions about treatment, and potentially predict outcomes. 

Given that deep learning (DL) algorithms, such as convolutional neural networks (CNNs) [4], recurrent neural networks (RNNs) [5], and Transformers [6], have demonstrated their effectiveness in processing EEG signals, we aim to implement a novel method to process EEG signals obtained from DOC patients and classify different DOC states. However, the general rule for training an effective deep learning model with superior generalization is that a larger training dataset usually leads to better model performance. Specifically, the datasets obtained from natural scenarios are typically characterized by large data sizes. In contrast, datasets collected from clinical applications often have small data sizes, presenting significant challenges for deep learning models. How to obtain a robust and effective DL model based on limited data size for supporting an accurate DOC state classification and improving neurorehabilitation treatment is a highly research-worthy topic. 

To overcome the challenge posed by limited data size to the DL models, several strategies, including data augmentation [7], transfer learning [8], and multi-task pre-training [9], are formulated to increase data volume for training effective DL models. Among those strategies, multi-task learning involves training a deep learning model on multiple datasets collected from different tasks. Considering the knowledge of underlying characteristics of brain electrical activity that might be common across brain disorders, we have reasons to believe the performance of the EEG-based DL model might be improved by training the model on the EEG datasets collected from various tasks. Despite this, we cannot ignore the fact that the model performance may be impacted by the data distribution shift when trained on multiple datasets. Specifically, because different EEG datasets are acquired from various sources, it is likely that these datasets possess different data distributions, leading to adverse risks such as degradation of model performance, difficulties of model transferring, and model overfitting. The key point of enhancing model performance affected by distribution shift is to enable the model to learn distribution-invariant features and is adaptable to new distribution encountered during training on other datasets. Regarding this issue, we propose a cross-distribution self-supervised (CDS) pre-training strategy based on contrastive learning to minimize the distribution gap between the data distribution of different EEG datasets. Several open-source EEG datasets are utilized as the dataset of the pretext task to pre-train the model, and the EEG dataset for DOC state classification is used as the dataset of the target downstream task to fine-tune the model. 

The main contributions are depicted as follows: (1) we propose a novel multi-task pre-trained Transformer model (MutaPT) for classifying DOC states using EEG data. Considering the fact that the limited data size of the EEG dataset challenges the model performance of the DL model, we collect multiple open-source EEG datasets to expand the data size for pre-training the MutaPT. (2) we propose a CDS pre-training strategy to learn distribution-invariant features and enhance the model generalization affected by data distribution shifts between multiple EEG datasets. (3) Three open-source EEG datasets are utilized to pre-train the MutaPT. A dataset for DOC state classification is used to evaluate the corresponding model performance. A comparison study is conducted to show the effectiveness and superiority of the MutaPT model. 

## 2. Materials and Methods

### 2.1. Three Open Source Datasets

The specific illustration of the three open-source datasets is depicted as follows:

(1) A database for emotion analysis using physiological signals (DEAP) [10] is adopted to perform the emotion recognition task. Thirty-two participants joined the data collection experiments, each with 32 EEG channels at 512 Hz. The data for each participant can be represented by a matrix of video/trail×channel×length. The number of trails, channels, and lengths equals 40, 32, and 8064, respectively. We divided the EEG data of DEAP into four categories: high valence-high arousal (HVHA), high valence-low arousal (HVLA), low valence-high arousal (LVHA), and low valence-low arousal (LVLA);
(2) We also utilize the dataset collected by the Brain-like Computing and Machine Intelligence Laboratory at Shanghai Jiao Tong University (SJTU): SJTU Emotion EEG Dataset (SEED) [11] and SEED-IV [12]. Both of the two datasets contain EEG data of 15 subjects. The data for each participant in SEED can be represented by a matrix of session×channel×N. The number of sessions and channels equals 3 and 62, respectively. Three label categories are included in the SEED dataset: positive, negative, and neutral. The data for each participant in SEED-IV can be represented by a matrix of session×trail×channel×length. The number of sessions, trails, and channels equals 3, 24, and 62, respectively. Four label categories are included in the SEED-IV dataset: happy, sad, fear, and neutral.

Besides the three open-source datasets, an EEG dataset for DOC state classification was acquired from the Affiliated Rehabilitation Hospital of Nanchang University. The data acquisition was conducted in accordance with the Declaration of Helsinki and was approved by the Ethics Committee of the Affiliated Rehabilitation Hospital of Nanchang University (2020-137; approved 28 June 2020). For the EEG dataset of DOC state classification, we initially assessed 352 patients for eligibility. Out of these, 301 met the inclusion criteria and were eligible for the study. However, 16 patients declined to participate, leaving 285 patients who were randomized. These 285 patients were then randomly assigned into three groups: 38 patients with emergence from the minimally conscious state (EMCS), 126 patients with emergence from the minimally conscious state (MCS), and 121 patients with vegetative state (VS). For convenience’s sake, we name the dataset DOC. The data for each participant can be represented by a matrix of channel×N, and the number of channels equals 32. The inclusion criteria for the study were as follows: (1) patients diagnosed with EMCS, MCS, vs. through coma recovery scale-revised (CRS-R); (2) patients in a stable condition; (3) patients with a duration of more than 1 month; and (4) patients with no signs of improvement in consciousness for more than 1 month. The exclusion criteria were as follows: (1) patients with a history of neurological diseases or psychiatric disorders; (2) patients taking drugs or undergoing any other treatments that may affect cortical excitability; (3) patients with epilepsy or frequent uncontrolled spontaneous movements; and (4) patients with pacemakers, arterial clips, or other metal implants in the body. Table 1 gives a simple illustration of the EEG datasets. 

### 2.2. Data Organization and Normalization

**Figure 1:**
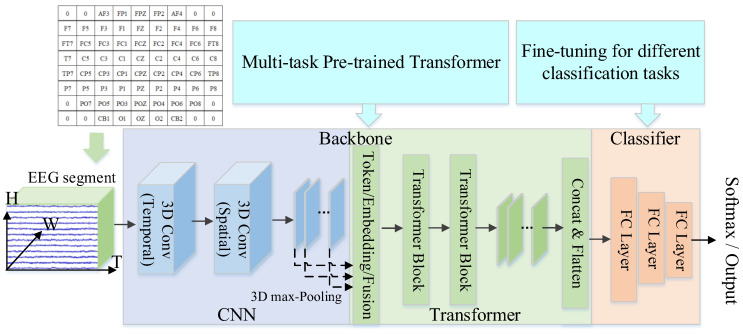
Model architecture of the MutaPT model.

Due to different datasets containing different numbers of EEG channels, we built a three-dimensional data structure to organize the data and unify the model input. More specifically, the original EEG trials are divided into Ts long segments without overlapping, and every segment is assigned with the label of the original trial. Suppose an original EEG segment is represented as $\mathrm{Seg}\in R^{m\times rT}$, where m and r denote the number of electrodes and the sampling rate of the raw EEG signals, respectively. Then, to preserve the spatial structure information of the electrode location, we organized the two-dimensional segment into a three-dimensional array according to a compact two-dimensional map. The map is shown in the top left corner of Figure 1; a value of zero indicates that the channels’ signals are unused. The three-dimensional (3D) array can be represented as $X\in R^{h\times w\times rT}$, where *h* and *w* are the height and width of the compact two-dimensional (2D) map, respectively. 

### 2.3. Model Architecture

#### 2.3.1. Backbone Network

Figure 1 shows the model architecture of the MutaPT. As shown in the figure, the backbone consists of a CNN and a Transformer. For the CNN, we first use a 3D convolutional layer to convolve the 3D input data in a channel-wise manner. More specifically, the layer applies *K* 3D filters to the input data, each filter $F\in R^{h_{1}\times w_{1}\times d_{1}}$ moves 3-directionally (width (W), height (H), temporal (T)) to calculate the feature. We set $h_{1}$, $w_{1}$ and $d_{1}$ to 1, 1, and 25, respectively. The strides of the filter along the three directions are set to 1. The output of the 3D convolutional layer can be represented by $X^{1}\in R^{h\times w\times rT\times K}$, which can be regarded as *K* 3D feature maps. After that, we use another 3D convolutional layer to convolve the $X^{1}$, and the layer also consists of *K* 3D filters. The size of each filter is $h_{2}\times w_{2}\times d_{2}$, where $h_{2}$, $w_{2}$ and $d_{2}$ are set to 5, 5, and 1, respectively. The strides of the filter along the three directions are set to 1. The output of the second 3D convolutional layer can be represented by $X^{2}\in R^{h\times w\times rT\times K}$. A combination of batch normalization and ELU activation is utilized to process the $X^{2}$. After that, a 3D max-pooling layer is adopted to down-sample the $X^{2}$ along the temporal dimension. The size of the pooling kernel and the stride are set to (1, 1, 75) and (1, 1, 15), respectively. The output of the 3D max-pooling layer can be represented by $X^{3}\in R^{h\times w\times \left(\frac{rT}{15}\right)\times K}$. A dropout layer is applied to process the $X^{3}$ with a dropout rate of 0.5. The final output of the CNN part is represented by $X^{CNN}\in R^{h\times w\times l\times K}$ and $X^{scale}\in R^{l}$, where *l* is the length of the vector outputted by 1DCNN, and its value equals to $\frac{rT}{15}$. 

For the Transformer part, it contains an embedding layer, two Transformer blocks, and a 3D max-pooling layer. The embedding layer is responsible for tokenizing the $X^{CNN}$ and embedding temporal and spatial information into $X^{CNN}$. Specifically, as shown in Figure 1, the dashed line with an arrow denotes that we split the $X^{CNN}$ into *K* 3D feature maps and iteratively feed a 3D feature map into the embedding layer. A single 3D feature map of $X^{CNN}$ is represented by $X_{i}^{CNN}\in R^{h\times w\times l}$. The embedding layer firstly reshapes the $X_{i}^{CNN}$ into 2D, and then tokenize it into *M* tokens, where *M* equals h×w, and a single token is denoted by $x_{j}\in R^{l}$. After that, the embedding layer performs two types of position embedding operations: temporal position embedding (TEPE) and spatial position embedding (SPPE) to embed the temporal information and spatial information into $X_{i}^{CNN}$, respectively. The formula for calculating the TEPE is listed as follows:

(1) TEPE(pos, 2k)=sin(pos/10,000^(2k/M)),

(2) TEPE(pos, 2k+1)=cos(pos/10,000^(2k/M)),

where *pos*∈[0, *L*−1] denotes the time point of the value in $x_{j}$, and *k*∈[0, *M*−1] means embedded dimensionality. The cosine distance between Cartesian coordinates of EEG channels is chosen to generate spatial information. Specifically, the Cz electrode is selected as the reference electrode; the SPPE information for each token calculates the cosine distance between the Cz electrode and other electrodes. The formula of the SPPE is given as follows:

(3) $$\mathrm{SPPE}\left(k,\;\mathrm{Cz}\right)=\frac{P_{Cz}\cdot P_{k}}{\left\|P_{Cz}\|\|P_{k}\right\|}$$

where $P_{Cz}$ and $P_{i}$ indicate the Cartesian coordinates of the Cz electrode and the *k*-th electrode, respectively. From formula (3), we know that different EEG electrodes have different SPPE values. To embed the spatial information into $X_{i}^{CNN}$, we broadcast the SPPE value of a single electrode into a vector represented by $x_{sp}^{k}\in R^{l}$. The output of the SPPE module is denoted by $x_{sp}\in R^{M\times l}$. $Z\left(X_{i}^{CNN}\right)$ represents the output of the embedding layer, which is calculated as below:

(4) $$Z\left(X_{i}^{CNN}\right)=X_{i}^{CNN}+x_{te}+x_{sp}$$

The components of the Transformer block are listed in sequence: a Multi-head Self-Attention module, a dropout layer, a normalization layer with residual connection, a feed-forward neural network (FFN), a dropout layer, a normalization layer with residual connection, and a concatenate layer followed by flatten operation. Due to the Transformer block working on fixed-length input sequences and maintaining the same input shape across the layers, the output of the Transformer block is represented by $X_{i}^{TF}\in R^{h\times w\times l}$. *K* 3D feature maps are concatenated by the concatenate layer, and the formula is given as follows:

(5) $$X^{B}=Flatten\left(Concat\left(X_{i,1}^{TF},\;\ldots ,\;X_{i,K}^{TF}\right)\right),$$

where $X^{B}$ denotes backbone output. 

#### 2.3.2. Classifier

Three fully connected layers are employed as the classifier module, which outputs an *N*-dimensional vector after the softmax function. The first fully connected layer takes $X^{B}$ as input, applies a ReLU activation function, and outputs 512 units. The second fully connected layer then takes the 512-unit output from the previous layer, applies ReLU activation, and outputs 256 units. Since different tasks have a different number of categories for classification, the number of units in the last fully connected layer is not fixed. The number of units is set as the same as the number of categories for the specific task that the model is being trained to perform. 

### 2.4. Model Training Strategy

**Figure 2:**
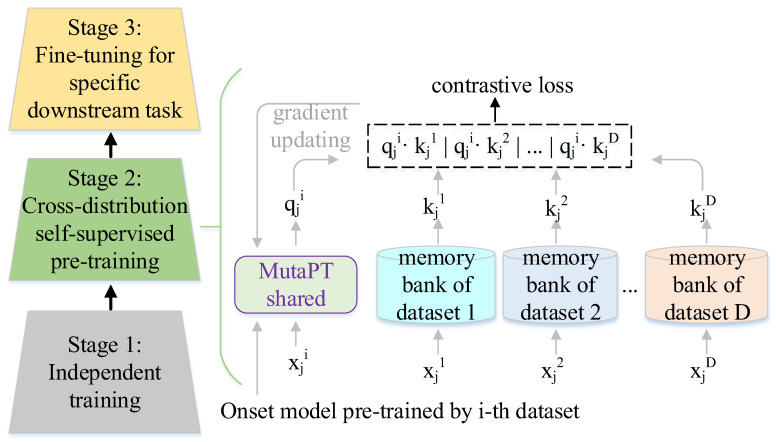
Pipeline of three-stage pre-training strategy.

We propose a three-stage pre-training strategy to train the MutaPT model on multiple open-source EEG datasets. Figure 2 shows the pipeline of the three-stage pre-training strategy, containing independently supervised pre-training, cross-distribution self-supervised (CDS) pre-training, and fine-tuning for target downstream tasks. The independent supervised pre-training phase is responsible for generating model weights. This involves pre-training the MutaPT model to fit the data distribution of each downstream task in a supervised manner. Thus, we can obtain a pre-trained model for each dataset; that is, the number of models is equal to the number of datasets. The CDS pre-training starts by using the MutaPT model trained by the dataset of the target downstream task as an onset model and then tries to learn robust representations that can effectively handle distribution differences in a self-supervised way. The fine-tuning for the target downstream task aims at adjusting model weights to optimize the model performance for the target downstream task. It is noteworthy that the CDS pre-training optimizes the model weights of the shared part of the MutaPT, and the fine-tuning tries to optimize the model weights of the classifier. 

#### 2.4.1. Independent Supervised Training 

Every dataset can be regarded as a dataset collected from a specific downstream task. Suppose we have *D* datasets collected from *D* downstream tasks. The independent supervised training aims at training the MutaPT on each dataset separately. Since every data processing for each dataset is regarded as a classification task, cross-entropy is used as the loss function to train the MutaPT model of each corresponding downstream task. The corresponding formula is depicted as follows:

(6) $${ℒ}_{i}=-\frac{1}{N_{b}}\sum_{j=1}^{N_{b}}\sum_{k=1}^{C}ylog\left(\hat{y}\right),\;i\in \left[1,\;D\right]$$

where *C* means the number of categories for the specific task, y and $\hat{y}$ are the ground truth and predicted label, respectively. $N_{b}$ represents the number of samples in a batch. 

#### 2.4.2. Cross-Distribution Self-Supervised Pre-Training 

The CDS pre-training strategy is proposed to tackle the challenge of the model performance being affected by data distribution shifts in multi-task learning. More specifically, due to the generalization capability of DL models being impacted by limited data availability, we hope to utilize the data samples collected from other downstream tasks to train the DL mode. This method might be effective for enhancing the model generalization as the underlying characteristics of brain activity are common across different downstream tasks. The key point of learning the underlying characteristics is to enable the model to learn robust representations that can effectively handle distribution differences in a self-supervised manner. This is performed by making relatively nearby cross-distribution samples closer while keeping dissimilar samples further. For convenience’s sake, we define the nearby cross-distribution samples and dissimilar samples as positive pairs and negative pairs, respectively. We first try to identify positive and negative cross-distribution pairs by computing the similarity distribution between query vectors and key vectors. We then optimized the MutaPT model by maximizing the distances between negative pairs and minimizing the distances between positive pairs. This enables us to develop a shared MutaPT model that can effectively generalize across all datasets used for training.

The *D* MUTAPT models trained in the phase of independent supervised training can be regarded as *D* feature extractors, and we use *F^i^*(·) (i∈[1, D]) to represent the *i*-th feature extractor. It is worth noting that the classifier part is removed from the MutaPT model. *D* memory banks, i.e., $V^{i}=\left[v_{1}^{i},v_{2}^{i},\;\ldots ,v_{N_{i}}^{i}\right],\;i\in \left[1,\;D\right]$, are initialized from the *D* datasets with the corresponding feature extractor $F^{i}\left(\cdot \right)$. $N_{i}$ represents the sample number of the *i*-th dataset used for training the model. In each iteration, we use $x_{j}^{i}$ to represent the *j*-th EEG segment belonging to the *i*-th dataset in batch *B*. The backbone of the MutaPT model trained by the *i*-th dataset is selected as an onset model representing a pre-trained model for the target downstream task. We obtain query feature vectors $q_{j}^{i}$ by feeding $x_{j}^{i}$ into the onset model. It is noteworthy that the length of the query vector and the key vector is 256, as the last fully connected layer of the MutaPT backbone outputs 256 units. Given the query vectors, we first measure cross-distribution pairwise similarities $P_{j,j^{\prime }}^{i\to i^{\prime }}$ between the query vector $q_{j}^{i}$ and the vectors $k_{j^{\prime }}^{i^{\prime }}$ stored in the $i^{\prime }$-th memory bank. The formula of the cross-distribution pairwise similarities is listed as follows:

(7) $$P_{j,j^{\prime }}^{i\to i^{\prime }}=\frac{\exp\left(\frac{{\left(k_{j^{\prime }}^{i^{\prime }}\right)}^{T}q_{j}^{i}}{\tau }\right)}{\sum_{j^{\prime }=1}^{N_{i^{\prime }}}\exp\left({\left(k_{j^{\prime }}^{i^{\prime }}\right)}^{T}q_{j}^{i}/\tau \right)},\;i\neq i^{\prime }$$

Then, the averaged entropy loss of the similarity distribution in a batch is calculated as follows:

(8) $${ℒ}_{CDS}=\frac{1}{\left|B\right|}(\sum_{i=1}^{D}\sum_{i^{\prime }=1}^{D}\sum_{j\in B}H\left(P_{j}^{i\to i^{\prime }}\right)),\;i\neq i^{\prime }\;$$

where H(·) means the entropy of the pairwise similarities between the query vector of the *j*-th segment of the *i*-th dataset in batch *B* and the $j^{\prime }$-th key vector of $i^{\prime }$-th memory bank. The formula is depicted as follows:

(9) $$H\left(P_{j}^{i\to i^{\prime }}\right)=-\sum_{j^{\prime }}^{N_{i^{\prime }}}P_{j,j^{\prime }}^{i\to i^{\prime }}logP_{j,j^{\prime }}^{i\to i^{\prime }},\;i\neq i^{\prime }$$

The features in the batch selected from the memory banks are updated with a momentum ρ for training the model smoothness. The formula is given as follows:

(10) $$\forall j\in B,\;v_{j}^{i}=\left(1-\rho \right)v_{j}^{i}+\rho k_{j}^{i},$$

#### 2.4.3. Fine-Tuning for Target Downstream Task

A random initialized classifier is added to the MutaPT backbone for performing the target downstream task. We first freeze the weights of the pre-trained MutaPT backbone to prevent them from being updated during the initial stages of training. This allows the model to leverage the learned common underlying characteristics across different downstream tasks from the pre-trained model without disturbing them. Then, we train the added classifier on the dataset of the target downstream task. This adapts the added classifier to the target task, leading to improved performance and fast convergence. 

## 3. Results

### 3.1. Evaluation Metrics

We evaluate the model performance by the metrics of accuracy (acc), specificity (spe), and sensitivity (sen). The corresponding formulas are listed as follows:

(11) $$\mathrm{Accuracy}\;=\frac{\mathrm{TP}\;+\;\mathrm{TN}}{\mathrm{TP}\;+\;\mathrm{TN}\;+\;\mathrm{FP}\;+\;\mathrm{FN}},$$

(12) $$\mathrm{Specificity}\;=\frac{\mathrm{TN}}{\mathrm{TN}\;+\;\mathrm{FP}},$$

(13) $$\mathrm{Sensitivity}\;=\frac{\mathrm{TP}}{\mathrm{TP}\;+\;\mathrm{FN}},$$

where TP, TN, FP, and FN denote true positive, true negative, false positive, and false negative, respectively. In the two-category classification, the data samples collected from patients and the data samples collected from health controls are regarded as positive samples and negative samples, respectively. In multi-category classification, the micro-averaged method is utilized to calculate the specificity and sensitivity as it weights each sample equally and generalizes in class imbalance. Leave-several-subjects-out (LSSO) cross-validation is used to form the training dataset, validation dataset, and testing dataset by using the proportion of 6:2:2. The three metrics are evaluated in a subject-wise manner. For segments belonging to the same subject, the majority rule is adopted to assign the final category to the subject by choosing the category with the most samples belonging to it. 

### 3.2. Implementation Details

At the interface level, MutaPT takes 3D EEG segments as input. For the convenience sake of data processing, we unify the time length of different datasets by splitting each data sample into several segments; the data shape of each segment is $X\in R^{8\times 9\times 2500}$. To reduce the burden of searching hyper-parameters for each single task, we adopt the same set of hyper-parameters to train the model in independent supervised training. The model is trained for 300 epochs using the Adam optimizer with an initial learning rate of 2 × 10^−4^. To reduce the time complexity, an early stopping strategy is utilized to train the model with a batch size of 8 in each iteration. 

### 3.3. Ablation Study

To validate the effectiveness of using the CDS pre-training strategy on the performance of the MutaPT model for classifying DOC states, we compared the model performance of the MutaPT with the model performance of the MutaPT without using the CDS (i.e., MutaPT_w/o CDS_), and the comparison results are shown in Table 2. For MutaPT_w/o CDS_, it achieved an average accuracy of 82.9% with a standard deviation of 5.4%. The specificity and sensitivity were recorded at 90.6% ± 7.7% and 80.8% ±7.4%, respectively. In comparison, the MUTAPT model demonstrated slightly higher performance. It obtained an average accuracy of 85.7% with a standard deviation of 2.9%. The specificity and sensitivity for MUTAPT were reported as 89.6% (±6.7%) and 82.1% (±5.8%), respectively. The experimental results demonstrate the effectiveness of the CDS pre-training strategy in addressing the challenge of model performance being affected by data distribution shifts in multi-task learning. The improvements in the MutaPT model’s performance suggest that this strategy may enable robust representations, effectively handling distribution differences between datasets of different tasks.

### 3.4. Comparison Study

Table 3 shows the results of comparing the model performance of the MutaPT with baseline models. Four classic deep learning models (EEGNet [13], DeepConvNet [14], ShallowConvNet [14], and EEG-Conformer [15]) for processing EEG data are selected as baseline models. It is worth noting that the baseline models are trained on a single dataset for the corresponding downstream task. The MutaPT model is utilized to classify the testing data of each dataset. Compared with the baseline models, the MutaPT demonstrates the highest average accuracy across all datasets. For specificity, which measures the model’s ability to correctly identify negative instances, MutaPT exhibits the highest average specificity for most datasets, with values ranging from 62.1% to 89.6%. The experimental results show that our multi-task model outperforms these models, indicating that the multi-task pre-training method based on datasets of multiple downstream tasks is beneficial for improving model performance. From the results, we can conclude that the performance of the MutaPT model can be enhanced through multi-task learning by integrating multiple EEG datasets from various tasks. This supports our hypothesis that using data samples collected from other downstream tasks to train the deep learning model can mitigate the impact of limited data availability on the model’s generalization capability.

## 4. Discussion

DOC represents a prevalent clinical condition involving abnormalities in brain function leading to a diminished level of consciousness. In clinical practice, EEG has been integrated into assessments of DOC patients with the aim of augmenting information sources associated with patients’ levels of consciousness, reducing misclassification rates, and exploring new prognostic factors [16,17]. Guidelines for DOC diagnosis recommend various instrument evaluations, with EEG emerging as more economical and readily applicable at the bedside. Over recent decades, the pivotal role of EEG in diagnosing and prognosticating DOC patients has become increasingly evident. Our research is also EEG-based, aimed at further exploring more efficient and accurate classification methodologies. We intend to amalgamate EEG data and incorporate techniques such as DL-based multi-task learning and model pre-training to elevate the performance of classifying consciousness disorders.

By investigating the previous works, we found that the performance of deep learning models for classifying disorders of consciousness may be hindered by the limited size of available data. To overcome the challenge posed by limited data size in clinical scenarios to the DL models, several strategies, including data augmentation, transfer learning, and multi-task pre-training are formulated. Among those strategies, multi-task learning technology has recently become a new advanced paradigm of deep model training that establishes state-of-the-art performance for specific tasks. Multi-task learning involves training a deep learning model on multiple datasets collected from different tasks. For example, 12-in-1 [18] trains the DL model on 12 different datasets from various task categories, yielding better model generalization performance compared to training the model independently on a single dataset. The model training strategy calculates task-specific loss in turn and updates the model parameters for each downstream task. 

Inspired by the work of 12-in-1, the motivation of this study is to implement a deep learning method trained on several open-source EEG datasets to alleviate the impact of limited data size on model performance. Furthermore, it must consider the fact that the model performance may be impacted by the challenge of data distribution shift when trained on multiple datasets. Contrastive learning provides a feasible approach to addressing the challenge of data distribution shift. For example, some studies focus on designing DL models to deal with issues including inter-subject variability and limited data size of EEG signals. Those issues challenge the performance of DL models for specific tasks. For instance, Shen et al. proposed a Contrastive Learning method for Inter-Subject Alignment (CLISA) to tackle the cross-subject emotion recognition problem [19]. Kostas et al. use a self-supervised training objective to learn compressed representations of raw data signals for brain–computer interface (BCI) classification and adopts a single pre-trained model to model completely novel raw EEG sequences recorded with differing hardware and different subjects performing different tasks [20]. In this context, we propose a novel multitask pre-trained transformer model that integrates CNN and transformer architectures, aiming to enhance the efficiency and accuracy of DOC state classification. 

The experimental results show the effectiveness of the CDS pre-training strategy in addressing the challenge of model performance being affected by data distribution shifts in multi-task learning. The improvements in the MutaPT model’s performance suggest that this strategy may enable robust representations, effectively handling distribution differences between datasets of different tasks. In addition, we can also conclude that the performance of the MutaPT model can be enhanced through multi-task learning by integrating multiple EEG datasets from various tasks. This supports our hypothesis that using data samples collected from other downstream tasks to train the deep learning model can mitigate the impact of limited data availability on the model’s generalization capability. 

Our study has several limitations. First, additional datasets are needed to increase the data volume and further validate the effectiveness of the proposed method. Second, inspired by more advanced contrastive learning methods, such as MoCo and its updated versions, we can refine the CDS training strategy to train the model on multiple EEG datasets. Third, this is a preliminary study on using deep learning models to classify states of disorders of consciousness (DOC). Further research should be tried to explore how the model can be used to provide auxiliary treatment for DOC patients in real-world settings. 

## 5. Conclusions

In this study, we propose a multi-modal pre-trained Transformer model (i.e., MutaPT) to perform an EEG-based DOC state classification task. The primary innovations include two aspects: (1) utilizing multiple EEG datasets to expand data size for pre-training the MutaPT, aiming at overcoming the challenge of model performance impacted on limited data availability; (2) proposing CDS pre-training strategy based on contrastive learning to enhance the model generalization affected by data distribution shift between multiple EEG datasets. Extensive experiments on four EEG datasets show the superiority of our MutaPT over several DL models for EEG classification. These results also support the feasibility of using multiple EEG datasets from different task domains to improve the generalization of the MutaPT model. Additionally, the CDS pre-training strategy effectively mitigates the impact of data distribution shifts on model generalization.

## Figures and Tables

**Table 1:** A simple illustration of four EEG datasets for training the MUTAPT model and performing EEG-based downstream tasks.

Dataset	Number of Patients	Data Shape	Category
DEAP	32	40 × 32 × 8064	4 (HVHA, HVLA, LVHA, LVLA)
SEED	15	3 × 62 × N	3 (positive, negative, and neutral)
SEED-IV	15	3 × 24 × 62 × N	4 (happy, sad, fear, and neutral)
DOC	285	32 × N	3 (EMCS, MCS, VS)

**Table 2:** Performance comparison between MUTAPT without the CDS and MUTAPT.

MUTAPT_w/o CDS_			MUTAPT		
acc	spe	sen	acc	spe	sen
82.9 ± 5.4	90.6 ± 7.7	80.8 ± 7.4	85.7 ± 2.9	89.6 ± 6.7	82.1 ± 5.8

**Table 3:** Model comparison between MUTAPT and classic baseline models.

		DEAP	SEED	SEED-IV	DOC
EEGNet	acc	52.3 ± 8.4	67.1 ± 10.0	52.5 ± 8.7	76.8 ± 5.7
	spe	54.6 ± 6.2	64.6 ± 6.9	53.8 ± 9.5	82.6 ± 4.7
	sen	51.9 ± 8.7	68.7 ± 8.2	51.4 ± 7.9	75.2 ± 4.4
DeepConvNet	acc	58.5 ± 7.8	63.0 ± 11.9	54.5 ± 11.9	73.2 ± 9.1
	spe	60.1 ± 8.3	63.1 ± 7.4	56.8 ± 10.5	71.5 ± 14.3
	sen	57.4 ± 7.6	65.3 ± 8.2	53.2 ± 7.9	72.5 ± 8.0
ShallowConvNet	acc	54.6 ± 7.1	69.5 ± 7.2	53.3 ± 6.8	87.1 ± 2.4
	spe	56.7 ± 5.8	71.5 ± 6.3	54.4 ± 9.6	91.5 ± 5.7
	sen	53.6 ± 8.2	68.6 ± 7.1	51.9 ± 8.1	85.7 ± 2.8
EEG-Conformer	acc	54.3 ± 7.1	62.3 ± 9.9	45.0 ± 5.7	78.6 ± 5.8
	spe	56.8 ± 8.6	63.5 ± 6.2	46.8 ± 6.7	80.7 ± 3.9
	sen	53.2 ± 6.4	61.6 ± 9.2	44.3 ± 7.2	76.7 ± 4.6
MUTAPT	acc	59.2 ± 3.5	72.6 ± 5.4	60.2 ± 5.4	85.7 ± 2.9
	spe	62.1 ± 6.8	73.9 ± 7.1	62.8 ± 4.1	89.6 ± 6.7
	sen	56.3 ± 5.9	71.3 ± 6.7	58.3 ± 8.2	82.1 ± 5.8

## Data Availability

The EEG data for DOC state classification that supports this study is not openly available due to ethical and privacy concerns and is available from the corresponding author (Y.B.) upon reasonable request.
